# Correction to “Re: “Palliative Care in Europe: Safeguarding Compassion Amidst Changing End‐Of‐Life Policies and Expanding Access to Medically Assisted Dying Position Statement on Behalf of the European Academy of Neurology, the European Federation of Neurological Associations and OneNeurology” by Rukavina Et al”

**DOI:** 10.1111/ene.70794

**Published:** 2026-10-20

E. Pucci , A. Solari , G. Mancardi , L. Provinciali , N. Ticozzi , and A. Padovani , “Re: “Palliative Care in Europe: Safeguarding Compassion Amidst Changing End‐Of‐Life Policies and Expanding Access to Medically Assisted Dying Position Statement on Behalf of the European Academy of Neurology, the European Federation of Neurological Associations and OneNeurology” by Rukavina Et al,” European Journal of Neurology 33, no. 9 (2026): e70758, 10.1111/ene.70758.PMC1358432542752942

The affiliations for Alessandra Solari were incorrect. It should have been: Territorial Ethics Committee Lombardy 1, San Raffaele Hospital, Milan, Italy; Fondazione IRCCS Istituto Neurologico Carlo Besta, Milan, Italy.

In addition, the ORCID number of Alessandra Solari (0000‐0001‐9930‐7579) was missing.

We apologize for these errors.

